# Slightly photo-crosslinked chitosan/silk fibroin hydrogel adhesives with hemostasis and anti-inflammation for pro-healing cyclophosphamide-induced hemorrhagic cystitis

**DOI:** 10.1016/j.mtbio.2024.100947

**Published:** 2024-01-05

**Authors:** Jie Yao, Yaoqi Chen, Xiang Zhang, Junfeng Chen, Cheng Zhou, Junhui Jiang, Hua Zhang, Kerong Wu

**Affiliations:** a Department of Urology, Translational Research Laboratory for Urology, Ningbo Clinical Research Center for Urological Disease, The First Affiliated Hospital of Ningbo University, Ningbo, Zhejiang, 315010, China; b State Key Laboratory of Fluid Power and Mechatronic Systems, Zhejiang University, Hangzhou, Zhejiang, 310027, China; c Research Institute of Smart Medicine and Biological Engineering, Ningbo University, Ningbo, Zhejiang, 315211, China; d Key Laboratory of Precision Medicine for Atherosclerotic Diseases of Zhejiang Province, The First Affiliated Hospital of Ningbo University, Ningbo, Zhejiang, 315010, China

**Keywords:** Hemorrhagic cystitis, Slightly photo-crosslinked hydrogels, Wet-adhesion, Hemostasis, Anti-inflammation

## Abstract

Cyclophosphamide is commonly used in the treatment of various cancers and autoimmune diseases, while concurrently imposing substantial toxicity on the bladder, frequently manifesting hemorrhagic cystitis. Intravesical interventions, such as hyaluronic acid supplementation, present a therapeutic strategy to reinstate bladder barrier function and alleviate the effects of metabolic toxicants. However, it remains a great challenge to achieve efficient cyclophosphamide-induced hemorrhagic cystitis (CHC) management with accelerated tissue repair owing to the low wet-adhesion, poor hemostasis, and acute inflammatory responses. To address these issues, a hemostatic and anti-inflammatory hydrogel adhesive of chitosan methylacryloyl/silk fibroin methylacryloyl (CHMA/SFMA) is developed for promoting the healing of CHC. The obtained hydrogels show a high adhesive strength of 26.21 N/m with porcine bladder, facilitating the rapid hemostasis within 15 s, and reinstate bladder barrier function. Moreover, this hydrogel adhesive promotes the proliferation and aggregation of SV-HUC-1 and regulates macrophage polarization. Implanting the hydrogels into CHC bladders of a SD rat model, they not only can be completely biodegraded in 14 days, but also effectively control hematuria and inflammation, and accelerate angiogenesis, thereby significantly promote the healing of bladder injury. Overall, CHMA/SFMA hydrogels exhibit rapid hemostasis for treating CHC and accelerate muscle tissue repair via angiogenesis and inflammation amelioration, which may provide a new path for managing severe hemorrhagic cystitis in the clinics.

## Graphical abstract

**Figure:**
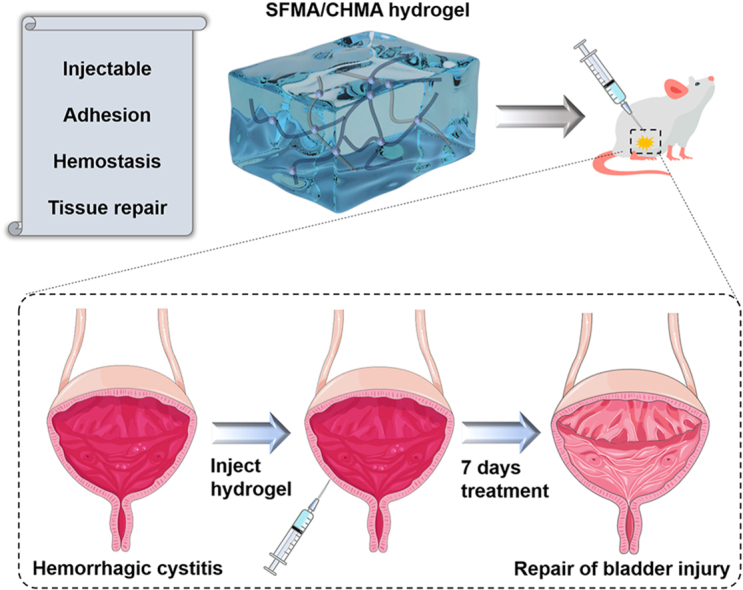

## 1 Introduction

Chemical hemorrhagic cystitis is a disorder characterized by diffuse inflammation and bleeding, frequently caused by various chemotherapy drugs such as cyclophosphamide and ifosfamide [[1], [2], [3]]. The severity of hematuria in chemical hemorrhagic cystitis varies from mild bleeding to life threatening persistent hemorrhage, requiring interventions like blood transfusion, medication, bladder perfusion, and hospitalization. Although interventional treatments such as vascular embolization, cystectomy and urinary diversion are effective, they often entail severe side effects. Intravesical instillations using hyaluronic acid, aluminum salt, or formalin are considered less aggressive and are usually typically administered before interventional approaches. However, these instillations lack efficacy in hemostasis, tissue repair and regeneration [[4], [5], [6], [7], [8], [9], [10]]. Consequently, there is an urgent need to explore innovative therapeutic options that are less invasive and offer enhanced hemostatic effects as well as tissue repair for hemorrhagic cystitis, which holds great scientific and clinical significance.

Hydrogels, characterized by a crosslinked three-dimensional polymeric network with excellent biocompatibility and a porous structure resembling the extracellular matrix (ECM), have become compelling candidates for bladder injury repair [[11], [12], [13], [14], [15]]. For instance, Rappaport et al. reported a thermosensitive hydrogel, composed of Pluronic F127, poly (ethylene glycol), and hydroxy propyl methyl cellulose, to treat interstitial cystitis with botulinum toxin-A [16]. The results indicated that intravesical instillation of such a hydrogel was safe, providing temporary efficacy lasting for a few weeks. Similarly, Li et al. developed sulfhydryl functionalized hyaluronic acid (HA-SH)/dimethyl sulfoxide (DMSO) hydrogels to attenuate cyclophosphamide (CYP)-induced bladder injury [17]. The intravesical instillation of HA-SH/DMSO hydrogels alleviated bladder toxicity from CYP administration by forming a protective structure similar like the glycosaminoglycan layer on the bladder wall. Despite the alleviation capability of current hydrogels for hemorrhagic cystitis, their effectiveness for severely hemorrhagic cystitis is limited due to significant weakening and dilution by urine. This limitation arises from wet tissue surfaces exposed to urine and continuous stress stimulation resulting from bladder contraction and dilation. It is desirable and yet a challenge to develop a hydrogel with strong wet-adhesion preformation to protect the injured tissue from urine. Moreover, a trade-off exists between strong wet-adhesion and biodegradability in vivo, where stiffer, more viscous materials exhibit better wet-adhesion, while softer, less viscous materials enable rapid biodegradability [18,19]. Therefore, a major challenge remains in designing hydrogels that can both adhere well to the wet bladder inner-surface and effectively biodegrade for wound repair and tissue regeneration.

To modulate the inflammatory responses associated with hemorrhagic cystitis, hydrogels with immunomodulatory functions are essential. Natural polymer hydrogels have been receiving growing attention due to their commendable biocompatibility and bioactivity. For example, chitosan has been extensively employed to regulate macrophage immunomodulation, creating an immune microenvironment with remarkable effects on tissue regeneration. Additionally, chitosan's abundance of primary amines allows it to serve as an adhesive surface on wet interfaces [20,21]. Despite these intriguing features, the primary obstacle for the biomedical application of chitosan lies in its poor solubility, low cellular affinity, and inadequate adhesive strength when used alone [22,23]. Therefore, collaborative efforts with other natural polymers are essential to overcome the inherent limitations of chitosan and maximize its potential in biomedical applications. In particular, silk fibroin (SF), a naturally occurring fibrous protein derived from Bombyx mori, has been identified as a promising enhancer for augmenting the efficacy of chitosan-based materials. SF contains substantial glycine-alanine repeat sequences, which confer robust mechanical properties to SF through a high propensity for *β*-sheet crystallization. SF with minimal inflammatory response renders it an ideal component for the construction of chitosan-based composites. While SF/chitosan composite hydrogels have found application as wound dressings to expedite wound healing [24,25], they have not been utilized in the treatment of hemorrhagic cystitis.

**Scheme 1:**
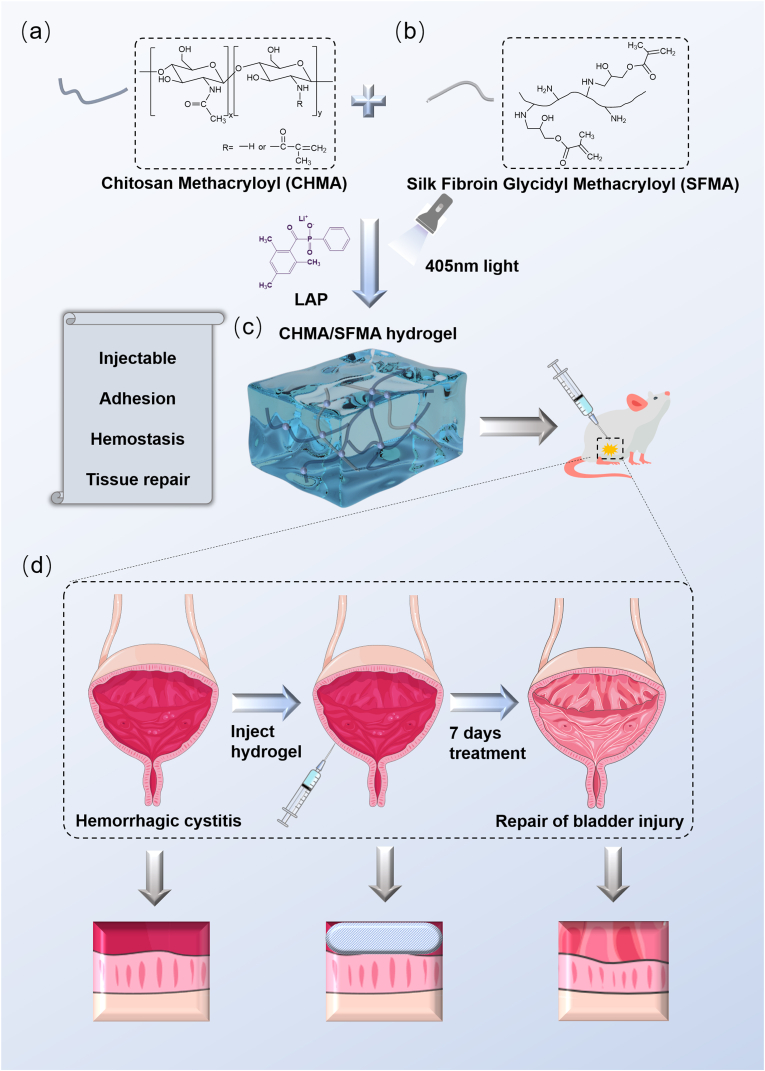
Schematic illustration to the preparation of the slightly crosslinked chitosan methylacryloyl/silk fibroin glycidyl methylacryloyl (CHMA/SFMA) hydrogel adhesives for bladder wound repair. (a–c) The chemical structures of CHMA (a) and SFMA (b), as well as network structures of crosslinked CHMA/SFMA hydrogels (c). (d) The utilization of CHMA/SFMA hydrogel adhesives for repairing hemorrhagic cystitis.

In this work, we present a slightly photo-crosslinked hydrogel adhesive comprising water-soluble chitosan methylacryloyl/silk fibroin methylacryloyl (CHMA/SFMA) with hemostatic and anti-inflammatory properties to promote the healing of cyclophosphamide-induced hemorrhagic cystitis (CHC) (Scheme 1a-c). The microstructures of the hydrogels were well-tuned to optimize wet-adhesion properties and biodegradability within the bladder. Porcine bladders and artificial urine were employed to mimic bladder micro-environments, revealing the prolonged protection for bladder tissues through high wet-adhesion capabilities. The adhesion strength of the optimized hydrogels on bladders reached up to 26.21 N/m. Furthermore, the CHMA/SFMA hydrogels facilitated the robust growth of human ureteral epithelial immortalized cells (SV-HUC-1) and induced the formation of cell micro-aggerates. These hydrogels exhibited a significant regulation of macrophage polarization, effective hemostatic ability, and appropriate biodegradability. Implanting CHMA/SFMA hydrogels into an SD rat CHC model revealed that the hydrogel adhesives regulate inflammatory effects, promote angiogenesis, and facilitate tissue regeneration for CHC treatment (Scheme 1d). This study affords a versatile CHMA/SFMA hydrogel as a promising candidate tissue-adhesive for rapid CHC healing.

## 2 Experimental section

### 2.1 Materials

Chitosan (CS, low molecular weight, degree of deacetylation >75 %, Sigma-Aldrich, 448869), glycidyl methacrylate (GMA, Sigma-Aldrich, 779342), methacrylic anhydride (MA, Sigma-Aldrich, 276685), lithium phenyl (2,4,6-trimethylbenzoyl) phosphinate (LAP, Sigma-Aldrich, 900889), sodium carbonate (Na_2_CO_3_, Shanghai Aladdin Bio-Chem Technology Co., LTD, S111737), lithium bromide (LiBr, Shanghai Aladdin Bio-ChemTechnology Co., LTD, L108931), CCK-8 kit (Beyotime, 40203ES60), TRITC Phalloidin (Solarbio, CA1610), 4 % paraformaldehyde (Solarbio, P1110), fetal bovine serum (FBS, Gibco, 10099–141), trypsin-EDTA (Gibco, 25200072), F-12 Ham's Kaighn's Modification (F12K, Solarbio, LA1320), RAW 264.7 Cell-Specific Culture Media (Pricella, CM-0190), lipopolysaccharides (LPS, Solarbio, L8880), Mouse TNF-alpha ELISA Kit (Proteintech, KE10002), penicillin streptomycin (Life Technologies, 15070063), regenerated silk fibroin (SF) was extracted from B. mori cocoons (Zhejiang Jiaxin Silk Co., LTD) according to a previous study with slight modifications. All other chemicals were used as received without further purification.

### 2.2 Synthesis and characterization of CHMA

Chitosan (CH, viscosity: 100–200 mPa s, degree of deacetylation ≈95 %) was dissolved in 2 wt% acetic acid solution to 1 wt% concentration. Methacrylic anhydride was added dropwise to this solution at a molar ratio of 1 anhydride per amino group. The reaction was conducted at 60 °C for 3 h. The resulting solution was then neutralized and diluted tenfold with 10 % (w/v) NaHCO_3_ solution. CHMA was dialyzed using a dialysis tube with cutoff molecular weight of 8–14 kDa against deionized water for 6 days to remove any unreacted reagents, followed by lyophilization. The obtained CHMA was analyzed by ^1^H NMR on a Bruker NMR (500 MHz) with D_2_O as the solvent. The Fourier Transform Infrared spectra (FTIR) were recorded on a Perkin-Elmer Spectrum One by the KBr pellets method. The measurement was carried out at 298 K, covering the range from 4000 to 500 cm^−1^. The degree of substitution (*DS*) of CHMA was calculated according to Equation:

$$DS\left(\%\right)=\frac{A_{H}\left(5.5\&5.8\right)/2}{A_{H}\left(2.5-4.2\right)/5}\times 95\%$$

where *A*_*H*_ (5.5 & 5.8) and *A*_*H*_ (2.5–4.2) were the area of methylene protons peak (H) at 5.5 and 5.8 ppm, and the ring protons peak of glucosamine (GlcN) residues at 2.5–4.2 ppm, respectively. Calculations based on ^1^H NMR results showed the degree of substitution of approximately 18.6 %.

### 2.3 Synthesis and characterization of SFMA

The SFMA solution was synthesized from SF and glycidyl methacrylate solution according to previously published method [26,27]. Briefly, 40 g of sliced cocoon was boiled in 1 L of 0.05 M Na_2_CO_3_ solution for 30 min at 100 °C to remove the sericin and then washed with distilled water (DW) several times. Subsequently, the degummed silk was dried at room temperature, and 20 g of it was dissolved in 100 mL of 9.3 M LiBr solution at 60 °C for 1 h 6 mL (424 mM) of GMA was added to the mixture and stirred at 300 rpm for 3 h at 60 °C. Then, the resulting solution was filtered through gauze and dialyzed against distilled water using 12–14 kDa dialysis tubes (Solarbio) for 4 days. Finally, the silk fibroin glycidyl methylacryloyl solutions were freeze-dried for 48 h. Lyophilized SFMA powder was stored at -80 °C for further use. The degree of methacrylation was defined according to the percentage of ε-amino groups of lysine in SF that are modified in SFMA. The signal from the protons produced by the aromatic amino acids in SFMA at 6.9–7.5 ppm was used to normalize each spectrum. The lysine methylene signals (2.8–2.95 ppm) of samples were integrated to obtain the areas. The degree of methacrylate substitution was determined by the following formula:

$$DS\;\left(\%\right)=\left(1-\frac{\text{Lys}\;\text{integration}\;\text{signal}\;\text{of}\;\text{SFMA}}{\text{Lys}\;\text{integration}\;\text{signal}\;\text{of}\;\text{unsubstituted}\;\text{SF}}\right)\times 100\%$$

### 2.4 Preparation of CHMA/SFMA hydrogels

To prepare the composite hydrogel precursor solutions, CHMA and SFMA were separately dissolved in ddH_2_O to achieve the desired concentrations. The CHMA and SFMA precursor solutions were then mixed in the desired ratio to achieve the desired composite composition, including 2%CHMA, 10%SFMA, 1%CHMA/5%SFMA, 1%CHMA/10 % SFMA, 2%CHMA/5%SFMA, and 2%CHMA/10%SFMA. A 0.025 % photoinitiator LAP was added to each precursor solution. After exposing to 405 nm UV light for 10 s, CHMA/SFMA hydrogels were successfully prepared. All polymer concentrations were presented as mass-to-volume ratios unless otherwise specified.

### 2.5 Microstructure characterization

The microstructures of the dehydrated samples were obtained using a scanning electron microscope (SEM, Phenom Pharos G2) after sputter coating with gold under vacuum.

### 2.6 Mechanical testing of CHMA/SFMA hydrogels

The mechanical properties of the hydrogels were assessed using a universal testing machine (SASCK, CMT1104, China) equipped with a 200 N load cell. For the 90° peeling test, a rectangular hydrogel sample measuring 30 mm × 15 mm was used. The test was conducted after a 15 min waiting period to ensure proper adhesion to the interfaces of porcine skin and porcine bladder, respectively. The derived mean and standard deviation values were based on at least three measurements.

### 2.7 Rheological characterization of CHMA/SFMA hydrogels

Rheological testing was conducted using a TA Instruments DHR-20 Rheometer with a 40 mm measuring plate at 25 °C. Frequency sweeps ranging from 0.1 to 100 rad⋅ s^−1^ were performed at a 1 % strain. Dynamic strain step tests were carried out by using 10 % and 1 % strain for 100 s each, repeated for five cycles. Shear rate sweeps from 0.1 to 100 s^−1^ were carried out to measure viscosity. For the photo-crosslinking process, the time sweep was conducted under 405 nm UV-light exposure at 10 Hz and 1 % strain until the storage modulus reached a plateau. The microstructures of hydrogels were analyzed by using rubber elastic theory. The approximate relationship between polymer network pore size (V) and modulus(G) is as follows:

$$G\approx \frac{K_{B}T}{V}$$

where *G* stands for storage modulus, *T* for temperature (298K), *K*_*B*_ for Boltzmann constant (1.38 × 10^−23^ J·K^−1^), and *V* for polymer network pore size.

### 2.8 Swelling and degradation experiments

For swelling analysis, the hydrogels, prepared in equal sizes (cylindrical shape with 8 mm in height and 10 mm in diameter) were placed into artificial urine solution at room temperature until the swelling equilibrium was reached. Subsequently, the surface moisture of the swollen hydrogels was absorbed, and they were weighed again (*W*_*t*_). The fresh hydrogels were weighed (*W*_*0*_). The swelling ratio (*SR*) of the hydrogels was calculated using the following formula (*n* = 3).

$$SR\;\left(\%\right)=\frac{W_{t}}{W_{0}}\times 100\%$$

For in-vitro degradation analysis, the hydrogels were immersed in artificial urine for 24 h to reach swelling equilibrium, and then the degradation behavior was monitored. All hydrogel scaffolds were placed in 1 mL of artificial urine containing 0.02 % sodium azide and 2 U/mL collagenase type I. The initial dry weight of the original hydrogel was recorded as *W*_*0*_. At specific time intervals, the hydrogel was removed, freeze-dried, and weighed, and the mass was recorded as *W*_*d*_. The degradation ratio (*DR*) of the hydrogels was calculated using the following formula:

$$DR\;\left(\%\right)=\frac{W_{0}-W_{d}}{W_{0}}\times 100\%$$

### 2.9 Cytocompatibility in vitro

SV-HUC-1 cell lines, generously provided by the Health Science Center of Ningbo University, were seeded on CHMA/SFMA hydrogel surfaces with a density of 5 × 10^4^ cells/mL in 24-well plates using Nutrient Mixture F-12 Ham's Kaighn's Modification supplemented with 10 % fetal bovine serum and 1 % penicillin/streptomycin. Medium was changed every other day. After 1, 3 and 5 days, the cell growth and proliferation on the hydrogels were assessed by using CCK-8 assay (Solarbio) following the manufacturer's protocol. In addition, F-actin and nuclei staining were stained using phalloidin and DAPI to visualize cellular morphology. Cellular imaging was conducted using a Laser Scanning Confocal Microscope (LSCM, Leica DMi8, Germany).

### 2.10 Macrophage polarization and enzyme-linked immunosorbent assay (ELISA)

In-vitro macrophage polarization was induced following established protocols [28]. Initially untreated RAW264.7 cells were designated as M0 macrophages. RAW264.7 cells (5 × 10^5^ cells/mL) were seeded in 24-well plates and allowed to adhere overnight before polarization. M1 macrophages were induced by stimulating RAW264.7 cells with LPS (10 ng/mL) for 24 h. Cytokine secretion was examined by using Mouse TNF-α (KE10002, Proteintech) following the manufacturer's protocols. Briefly, supernatants collected after 24 h of polarization were centrifuged at 500 g for 5 min to remove cellular debris. The cytokine levels were determined using the ELISA kit.

### 2.11 Hemolysis test

Hemolysis activity of 2%CHMA/10%SFMA hydrogel was evaluated following established procedures [29,30]. Red blood cells (RBCs) were collected from the rat whole blood samples by centrifugation. The RBCs were then diluted to 10 % v/v in saline and dispensed as per 500 μL in vials. 100 μL of 2%CHMA/10%SFMA hydrogel (*n* = 3) was added to each vial and incubated with RBCs at 37 °C for 1 h. 0.1 % Triton-X-100 and saline treatment (*n* = 3) were set as the positive and negative controls, respectively. After incubation for 1 h, the mixture was centrifuged 750*g* for 10 min, and the supernatant was transferred into a new 96-well plate. The absorbance at 545 nm was measured, and the hemolysis ratio (*HR*) was calculated using the following formula:

$$HR\;\left(\%\right)=\frac{{OD}_{Sample}-{OD}_{Nagetive}}{{OD}_{Positive}-{OD}_{Negative}}\times 100\%$$

where *OD*_*Sample*_, *OD*_*Nagetive*_ and *OD*_*Positive*_ stand for the optical density of hydrogel, saline, and 0.1 % Triton-X-100 solution.

### 2.12 Histocompatibility in-vivo

We employed SD rat model to assess hydrogel histocompatibility, following ethical approval from the Animal Ethics Committee of Ningbo University. The surgical procedure was conducted under aseptic conditions, involving a dorsal incision to expose the subcutaneous tissue. Subsequently, a 100 μL volume of hydrogel was carefully injected into the tissue using a needle at a predetermined implantation site. Post-implantation, rats were closely monitored for signs of infection or discomfort and provided with appropriate post-operative care. Skin tissue samples were collected at designated time points (DAY 3, DAY 7, DAY 14) for histological analysis. Hematoxylin-eosin staining was employed to assess tissue reactions. The experiment was performed with multiple experimental groups, including a control group, and repeated to ensure reproducibility and reliability of the results.

### 2.13 In-vivo hemostatic performance assessment

SD rat hemorrhaging liver model was utilized to evaluate the hemostatic efficacy of the investigated hydrogels. Briefly, after anesthetizing the SD rat, the liver was exposed through an abdominal incision and positioned over pre-weighed filter paper (*W*_*0*_). A controlled puncture was then created at a specific site using a sterile surgical blade. Subsequently, a 100 μL sample of the 2%CHMA/10%SFMA hydrogel was promptly administered to the bleeding site as a hemostatic sealant. Gauze and Surgical® Fibrillar™ were chosen as Control groups. The progression of bleeding from the liver was documented at specific time intervals using a digital camera. After 60 s, the filter paper was weighed again (*W*_*1*_) and the blood loss (*BL*) was calculated by the following formula:

$$BL=W_{1}-W_{0}$$

where *W*_*1*_*, W*_*0*_ stand for Initial mass of filter paper and mass of filter paper after blood absorption. The in vivo hemostasis experiment was replicated three times to ensure the consistency and reliability of the hydrogel treatment's effects.

### 2.14 CHC treatment in SD rat models

21 male Sprague Dawley rats weighing 250–280 g were used for CHC treatment. The rats were housed at room temperature with fixed limits of 55 ± 15 % relative humidity and 12 h/12 h light/dark cycle. Water and standard rat feed were administered ad libitum. All procedures related to animals were completed in accordance with national and international regulations about animal experiments. Without exception, all subjects survived until the end of the experiment, with no signs of illness such as weight loss, increased temperature, fur changes, or trembling.

The surgical procedures were mainly as follows. Firstly, open the abdominal cavity, locate the bladder, and inject 50 μL GEL or saline to bladder. Secondly, gently massage the bladder with hands to ensure distribution of the hydrogel along the bladder wall. Finally, suture the abdominal cavity and skin. The obtained bladder tissues were fixed in 4 % paraformaldehyde solution for further analysis. The experimental and control groups were organized as follows (n = 3).

**Table:** Surgical groups of SD rats Models and Treatment.

	SD rat Models	Treatment
BLANK	Saline	Saline
CTRL-DAY1	150 mg/kg CYP	
CTRL-DAY3		
CTRL-DAY7		
GEL-DAY1		Hydrogel adhesives
GEL-DAY3		
GEL-DAY7		

Here, CYP stands for cyclophosphamide, GEL and CTRL for hydrogel adhesives and control groups, respectively.

### 2.15 Histological and immunohistochemical analysis

After euthanizing the SD mice at each designated time point, the bladders were extracted and fixed in 4 % paraformaldehyde. Subsequently, the tissue samples underwent paraffin embedding and sectioning for hematoxylin and eosin (H&E) as well as Masson staining. Immunohistochemistry was employed to detect the expression of α-SMA, CD31, TNF-α, and IL-10.

### 2.16 Statistical analysis

All experiments were performed in three independent studies and data were expressed as means ± standard deviations. One-way analysis of variance (ANOVA) followed by Tukey's post hoc test or unpaired Student's t-test, two-tailed, was used to analyze the statistical significance where appropriate. Significant difference was considered at *p < 0.05, **p < 0.01 and ***p < 0.001.

## 3 Results and discussion

### 3.1 Preparation and characterization of CHMA/SFMA hydrogels

**Fig. 1:**
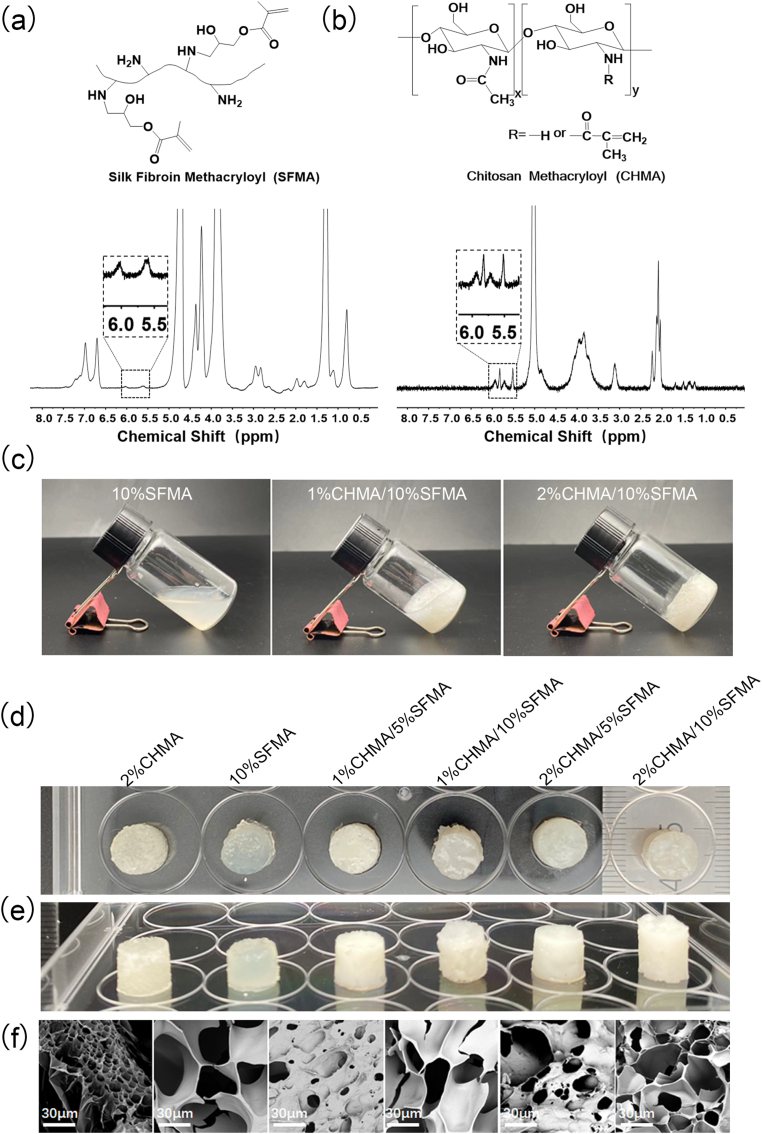
(a, b) ^1^H NMR spectra of SFMA (a) and CHMA (b), respectively. (c) Flow state of 10%SFMA, 1%CHMA/10%SFMA, 2%CHMA/10%SFMA hydrogel precursors. (d, e) Top-view (d) and front-view (e) digital images of CHMA/SFMA hydrogels with varies compositions. (f) SEM images illustrating the morphologies of freeze-dried hydrogels.

Hemostatic, anti-inflammatory, and biodegradable bladder adhesives based on slightly photo-crosslinked CHMA and SFMA hydrogels were prepared through photopolymerization under 405 nm blue light (10 mw·cm^−2^) with the photo-initiator LAP. Both CHMA and SFMA were synthesized by methacrylate substitution of the primary amine, with 18.6 % and 39 % substitution, according to ^1^H NMR measurements (Fig. 1a and b, Fig. S1, Supporting Information). A controlled photo-crosslinking time of 10 s facilitated partial chemical crosslinking of CHMA and SFMA chains, simultaneously establishing hydrogen bonding and electrostatic interactions. The states of hydrogels varied before photo-curing, particularly 2%CHMA/10%SFMA displaying higher viscosity and lower fluidity (Fig. 1c). Four composite formulations including 1%CHMA/5%SFMA, 1%CHMA/10%SFMA, 2%CHMA/5%SFMA and 2%CHMA/10%SFMA were produced for tissue-adhesive hydrogels, with 2%CHMA and 10%SFMA as control groups (Fig. 1d and e). All polymer concentrations were presented as mass-to-volume ratios unless otherwise specified. SEM analysis of freeze-dried hydrogels revealed interconnected porous microstructures (Fig. 1f), conducive to wound healing by aiding nutrient transmission and cell communication [[28], [29], [30], [31]]. The change of multiple porous structure was mainly attributed to a higher CHMA concentration in hydrogels, increasing crosslinking density. To mimic the bladder microenvironment, artificial urine solution was employed for swelling and degradation tests. Apart from a slight increase in the swelling ratio of 1%CHMA/5%SFMA, 2%CHMA/10%SFMA hydrogels to 104 % and 110 %, the swelling ratio of others decreased to 79 %, 84 %, 92 %, and 96 % for 2%CHMA, 10%SFMA, 2%CHMA/5%SFMA, 1%CHMA/10%SFMA (Fig. S2, Supporting Information). The reduction in hydrogel swelling was mainly attributed to the slight crosslinking, resulting in polymer runoff from the networks. In-vitro degradation revealed that the hydrogel completely degraded within 16 days in artificial urine containing 0.02 % sodium azide and 2 U/mL collagenase type I (Fig. S3, Supporting Information).

### 3.2 Rheological properties and adhesion assessment of CHMA/SFMA hydrogels

**Fig. 2:**
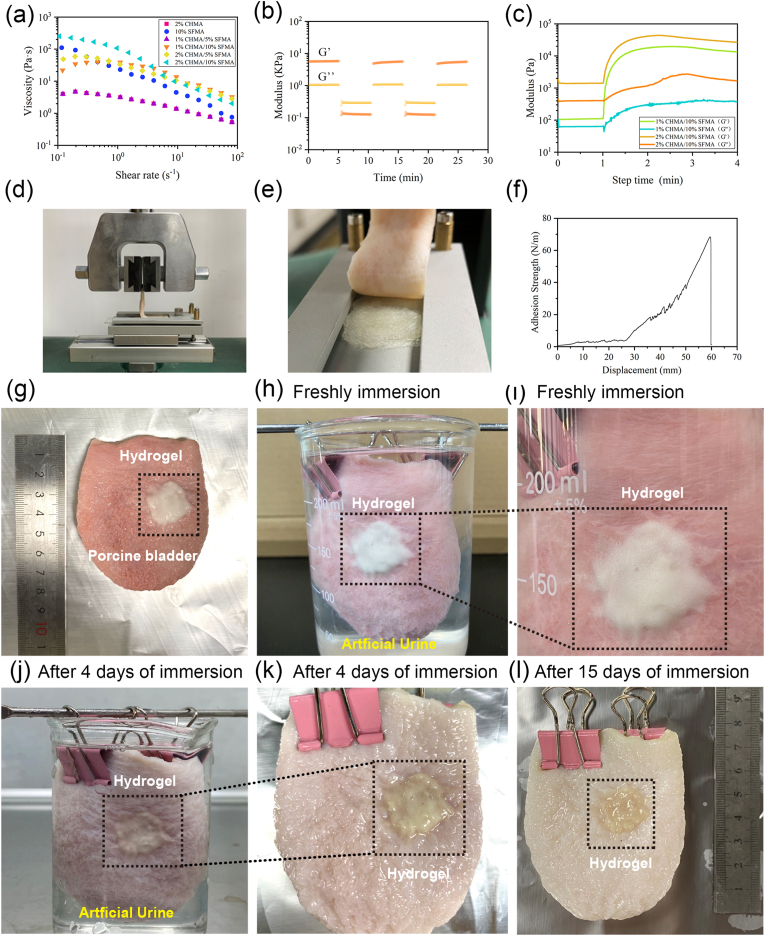
Rheological and tissue-adhesion characterization of CHMA/SFMA composite hydrogels. (a) Shear thinning characteristics of CHMA/SFMA hydrogel precursors with varying compositions illustrated through shear rate sweeps. (b) Reversible gel-sol transition and self-healing of 2%CHMA/10%SFMA pregels upon cyclic shearing at 1 % and 10 % strains at 25 °C. (c) Real-time monitoring of storage modulus (G′) and loss modulus (G″) of 1%CHMA/10%SFMA and 2%CHMA/10%SFMA hydrogels with 405 nm UV-light exposure. (d–f) Schematic of 90-degree peel test (d), and the representative images during the test process (e) and peel curve (f). (g) Representative photographs of 2 % CHMA/10 % SFMA hydrogels in-situ adhered onto the inner wall of the porcine bladder. (h) Bladders coated with hydrogels were immersed in artificial urine. (i) A detailed local magnification of the hydrogel attached to the surface of the porcine bladder. (j–l) Evaluation of the adhesion state of the hydrogel to the inner bladder wall on days 4 and 15 after exposure to artificial urine.

Rheology measurements were employed to evaluate the injectability and gelation properties of the CHMA/SFMA hydrogel system. As shown in Fig. 2a, CHMA/SFMA precursors exhibited a typical shear-thinning behavior, facilitating their injectability (Fig. S4, Supporting Information). Owing to the substantial presence of inherent hydrogen bonding and electrostatic interactions, the composite hydrogel precursors displayed a gel-like behavior, as indicated by the frequency sweep measurement (Fig. S5, Supporting Information). Moreover, due to these reversable interactions, the 2%CHMA/10%SFMA exhibited reversible gel-fluid transitions (Fig. 2b). This self-healing capability could be repeated many times, making it advantageous for in-situ bladder injection. Upon exposure to the 405 nm blue light, the storage modulus (G′) and the loss modulus (G″) of 1%CHMA/10%SFMA and 2%CHMA/10%SFMA exhibited a dramatic increase. The G’ increased by two orders of magnitude, confirming the formation of a stiff network structure (Fig. 2c), which was not conducive to biodegradation. Therefore, to achieve a slight photo-crosslinking network, the photo-polymerization time was kept within 10 s. The partially crosslinked networks were revealed using FI-TR spectrum, with a part of the double bond remaining in the network (Fig. S6, Supporting Information). According to rubber elastic theory, these slight-crosslinking CHMA/SFMA hydrogels exhibited nanostructures (Table S1, Supporting Information). Remarkably, the obtained CHMA/SFMA exhibited a robust mechanical loading capacity, enduring cyclical strains of up to 30 % compression. The hydrogels could instantly return to their original shape without cracking, exhibiting an impressive compressive stress of around 76 KPa at 30 % compressive strain (Fig. S7, Supporting Information). These results suggested that CHMA/SFMA hydrogels could adapt to the dynamic systolic-diastolic microenvironment of bladder.

To investigate the tissue-adhesion properties of the slightly CHMA/SFMA photo-crosslinked hydrogels, we conducted a 90° peeling experiment by using porcine skin (Fig. 2d and e), peeling off the porcine bladder completely. The maximum adhesion strength reached up to 68.42 N/m with porcine skin (Figs. 2f) and 26.21 N/m with porcine bladder (Fig. S8a, Supporting Information). Additionally, we meticulously prepared the porcine bladder by securing its interior with a clamp in a beaker and coating the inner wall of the bladder with a freshly prepared 2%CHMA/10%SFMA hydrogel. The bladder was then immersed in artificial urine and observed for 15 days. As shown in Fig. 2g–i, the hydrogels maintained prolonged adhesion to the bladder wall. Nevertheless, the adhesion strength markedly decreased to 13.74 N/m due to the swelling, degradation, and erosion of the hydrogels in artificial urine (Fig. S8b, Supporting Information). This enduring adhesion was primarily attributed to the synergistic effects of hydrogen bonds and electrostatic interactions within the 2%CHMA/10%SFMA hydrogel, forming a tight bond with the bladder tissue. It is worth noting that the variation in hydrogel adhesion to porcine bladder and porcine skin is mainly influenced by the distinct surface characteristics of the tissues, including differences in surface roughness, chemical compositions, and surface energy. The wet-adhesion positions the CHMA/SFMA hydrogel as a promising candidate for applications in hemostasis and wound healing within a urine-surrounding environment.

### 3.3 Biocompatibility of CHMA/SFMA hydrogels

**Fig. 3:**
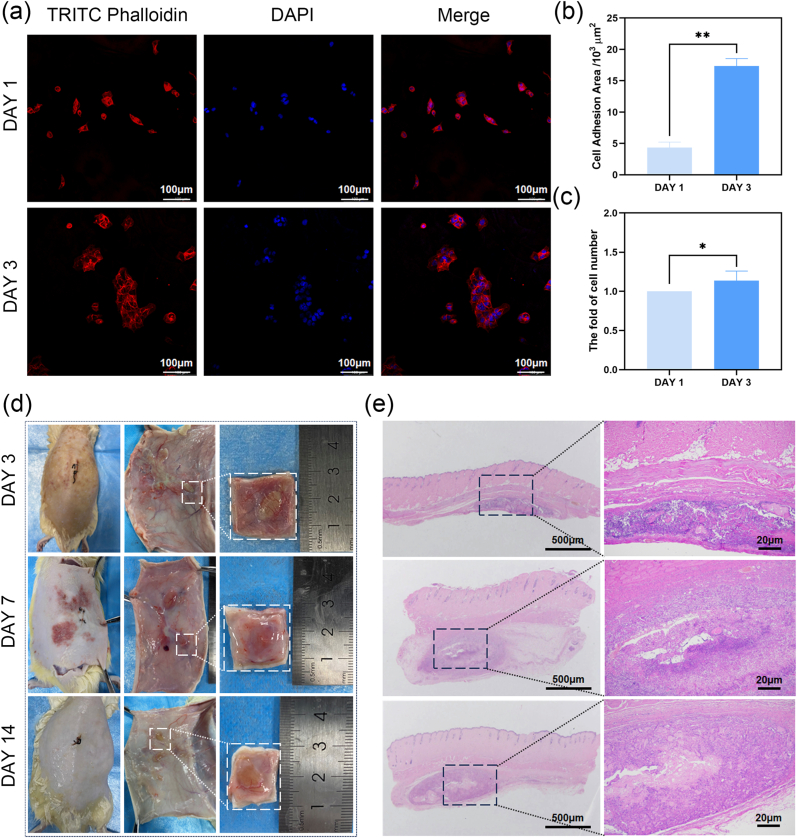
Biocompatibility assessment of 2%CHMA/10%SFMA hydrogels. (a) Representative fluorescent staining images depicting SV-HUC-1 morphologies on hydrogels at 1 and 3 days. (b) Quantification of the adhesion area of each microaggregate counted by the stained cellular nuclei at 1 and 3 days. (c) Quantitative analysis of SV-HUC-1 proliferation using CCK-8 assay. (d, e) Representative subcutaneous implantation (d) and H&E staining (e) images of 2%CHMA/10%SFMA hydrogels on DAY 3, DAY 7, DAY 14. *p < 0.05, **p < 0.01.

Biocompatibility is an essential prerequisite for implanting hydrogel in organs. SV-HUC-1 cells were chosen for their pivotal role in maintaining the integrity of the bladder and urinary tract epithelium. To assess the compatibility of these cells with 2%CHMA/10%SFMA hydrogels, cytoskeleton and nucleus staining were conducted to investigate the cellular morphology when cultured on the hydrogel substrates. As shown in Fig. 3a, cells seeded on the 2%CHMA/10%SFMA hydrogels exhibited robust adhesion, indicating excellent cytocompatibility. The adhesion area of a single cell spheroid grew from about 4.3 × 10^3^ to 17.3 × 10^3^ μm^2^ from day 1 to day 3 (Fig. 3b). Moreover, after 3 days of culture, the cell increased by 1.2-fold compared to day 1 (Fig. 3c). These results demonstrated that the 2%CHMA/10%SFMA hydrogels are ingenious cellular-responsive hydrogel systems, providing a specific microenvironment conducive to enhanced cell growth and interaction of SV-HUC-1.

Furthermore, subcutaneous implantation of hydrogel materials was carried out in a rat model to assess tissue responses and immune reactions. Hematoxylin-eosin staining analysis and meticulous examination of the hydrogel-tissue interface were employed. On days 3, 7, and 14, the back of Sprague-Dawley (SD) rats was surgically exposed for observation and imaging (Fig. 3d), and tissue section was collected and subsequently stained (Fig. 3e). On day 3 after transplantation, the hydrogel caused an obvious irritation reaction with the subcutaneous tissue, and large number of inflammatory cells clustering at the interface between the hydrogel and surrounding tissues. After 7 days of implantation, the inflammatory cells around the hydrogel decreased, and they basically disappeared after 14 days of implantation. No other obvious complications were observed throughout the process, confirming that the early inflammatory reaction is a normal response after in vivo implantation. These results indicated the good biocompatibility of the 2%CHMA/10%SFMA hydrogels, crucial for hemostatic applications and wound repair in the bladder.

**Fig. 4:**
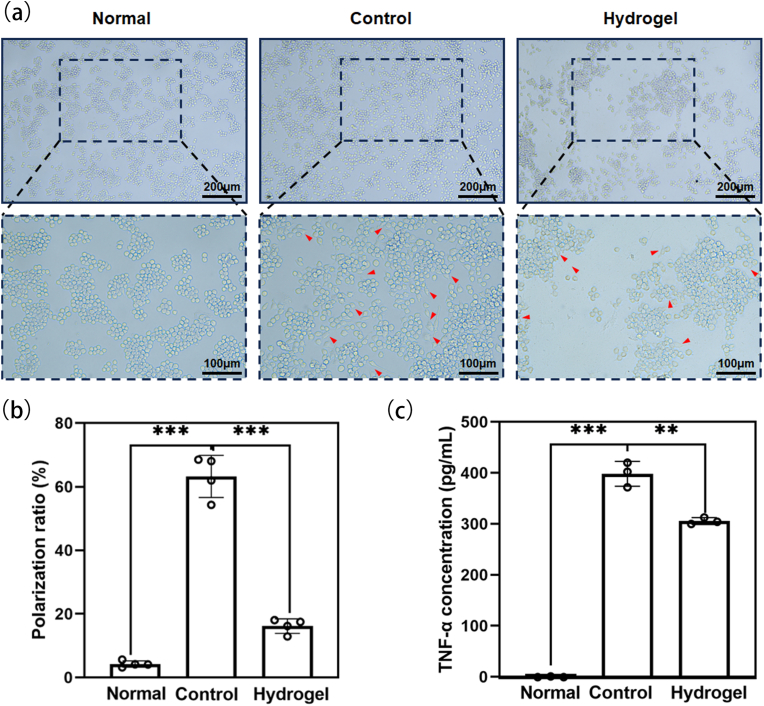
Morphological changes in RAW 264.7 cell line macrophages induced by 2%CHMA/10%SFMA hydrogel treatment in vitro. (a) Microscopic images capturing morphological alterations in RAW 264.7 cell line macrophages, with red arrows indicating polarized cells. Normal: RAW 264.7 cell line; Control: LPS-induced RAW 264.7 cell line; Hydrogel: 2% CHMA/10%SFMA hydrogel. (b) Quantification of macrophage polarization rate. (c) Measurement of TNF-α concentration in the supernatant of the three cell groups through ELISA assay. **p < 0.01, ***p < 0.001. (For interpretation of the references to color in this figure legend, the reader is referred to the Web version of this article.)

To elucidate the anti-inflammatory properties of 2%CHMA/10%SFMA hydrogels, a macrophage polarization assay was conducted. Macrophage cells, crucial in inflammation within wounds, play multifaceted roles in wound healing. The various phenotypes displayed by these cells are intricately linked to the processes of wound remodeling, scar formation, and fibrosis [31,32]. Balanced macrophage polarization critically influences the outcome of organ response in inflammation or injury [28,33]. Generally, macrophages activated by lipopolysaccharide (LPS) are labeled as M1-type macrophages. Macrophage M1 phenotype releases a large amount of TNF-α, IL-1β, IL-12, and IL-23 to counteract the external stimuli [31]. As shown Fig. 4a and b, RAW 264.7 cells strongly transformed into a spread M1 phenotype after LPS activation, while those on the 2%CHMA/10%SFMA hydrogel exhibited a spheroid M0 phenotype. Moreover, ELISA results revealed that 2%CHMA/10%SFMA hydrogels significantly reduced the release of TNF-α compared with the LPS-induced group (Fig. 4c). These results indicated an anti-inflammatory function of CHMA/SFMA hydrogel adhesives, primarily attributed to the combined contribution of composites.

### 3.4 Hemolysis, in-vivo hemostasis assessment and degradation of CHMA/SFMA hydrogels

**Fig. 5:**
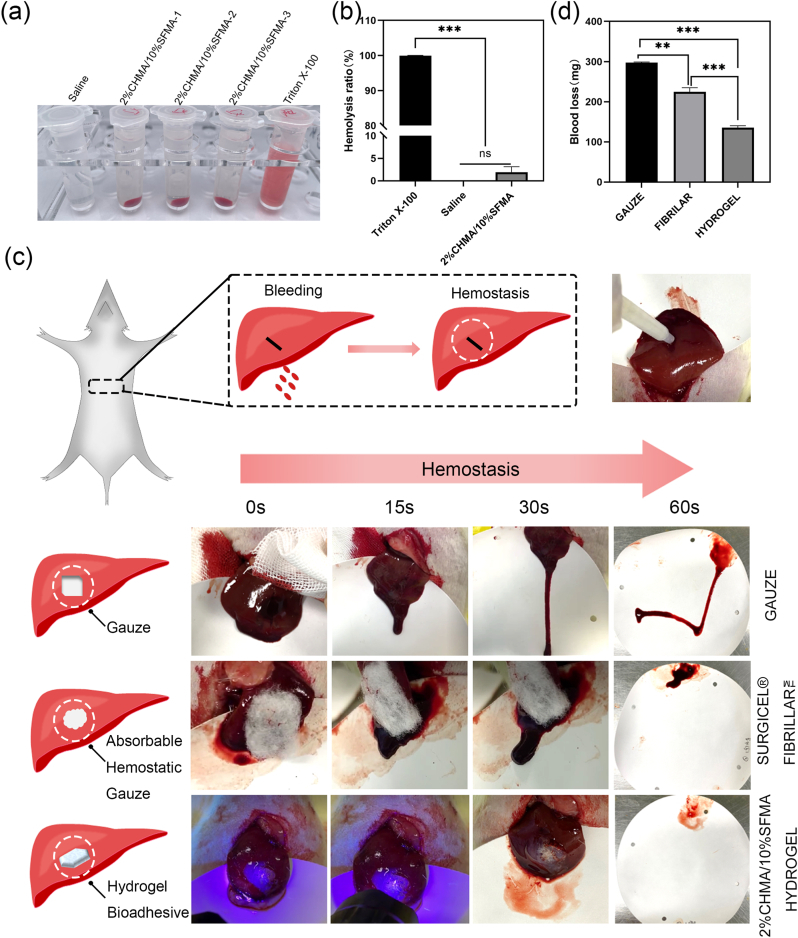
(a) In-vitro hemolysis test conducted on three parallel 2%CHMA/10%SFMA hydrogels. (b) Hemolysis ratio of negative control, positive control, and the three parallel hydrogel groups at 545 nm. (c) Schematic representation of liver hemostasis in SD rats, accompanied by photographs of wound treated with Gauze, surgicel® fibrillar™ and 2%CHMA/10%SFMA hydrogels. (d) Statistical analysis of blood loss for the three types of materials. **p < 0.01, ***p < 0.001.

To assess the hemocompatibility of the 2%CHMA/10%SFMA hydrogel, a hemolysis assay was conducted. As illustrated in Fig. 5a and b, the hemolysis ratio of the 2%CHMA/10%SFMA hydrogels was 1.94 %, significantly below the international standard threshold of 5 %, indicating their low hemolysis risk. Therefore, CHMA/SFMA hydrogels are blood-compatible and could be used for hemostasis and implantation in-vivo. The high wet-adhesion properties of CHMA/SFMA hydrogels on the bladder render them for hemostasis capabilities. To this end, we established a liver bleeding model in Sprague-Dawley (SD) rats (Fig. 5c). Commercially available gauze and Surgical® Fibrillar™, both wildly applied in clinical hemostasis, were employed as control groups. When the 2%CHMA/10%SFMA hydrogels were applied to the bleeding site, a rapid cessation of bleeding was observed, Complete hemostasis in the 2%CHMA/10%SFMA hydrogel group was achieved in 15 s, compared to 58 s and 30 s in gauze and Surgical® Fibrillar™ groups, respectively (Fig. S9, Supporting Information). The blood loss was only approximately 135.4 mg on the filter paper (Fig. 5d). In contrast, wounds managed with gauze and Surgical® Fibrillar™ continued to bleed for longer time, resulting in obvious bloodstain dispersion on the filter paper and a considerably higher blood loss ranging from 224.8 to 297.8 mg. These results demonstrated the impressive hemostatic property of the CHMA/SFMA hydrogel adhesives, primarily owing to their rapid photocuring and stable wet-adhesive properties.

The in-vivo degradation of hydrogel adhesives is critical within the bladder environment, mitigating the necessity for surgical removal and reducing the risk of complications, such as urinary tract obstruction. For degradation studies, bladder tissues were retrieved on the 14th day following the intravesical injection of the 2%CHMA/10%SFMA hydrogel in SD rats and subjected to H&E staining for microscopic examination. Remarkably, upon histological assessment, no residual presence or adherence of the hydrogels was observed on the bladder mucosa (Fig. S10, Supporting Information), indicating the complete degradation and clearance of the hydrogel by the 14th day post-injection within the bladder environment. These findings suggested the effective and timely degradation of the 2%CHMA/10%SFMA hydrogel adhesives, highlighting its potential suitability for bladder wound repair applications.

### 3.5 Treatment of CHMA/SFMA hydrogel adhesives for cyclophosphamide-induced hemorrhagic cystitis (CHC)

Cyclophosphamide (CYP)-induced hemorrhagic cystitis (CHC) is one of the most severe complications encountered in clinical application of chemotherapy. Clinical symptoms mainly include increased urinary urgency and frequency, hematuria ranging from microscopic to severe hematuria with clots and obstruction, and an inflammatory response [34,35]. Typically, hematuria becomes evident within 4 h following CYP administration, with significant alterations in bladder histology detectable by the 24-h mark [36]. The administration of CYP also results in substantial tissue infiltration by inflammatory cells and the upregulation of proinflammatory cytokines, such as IL-1β, IL-10, and TNF-α [37]. Therefore, the effective treatment of CHC is of increasing importance.

**Fig. 6:**
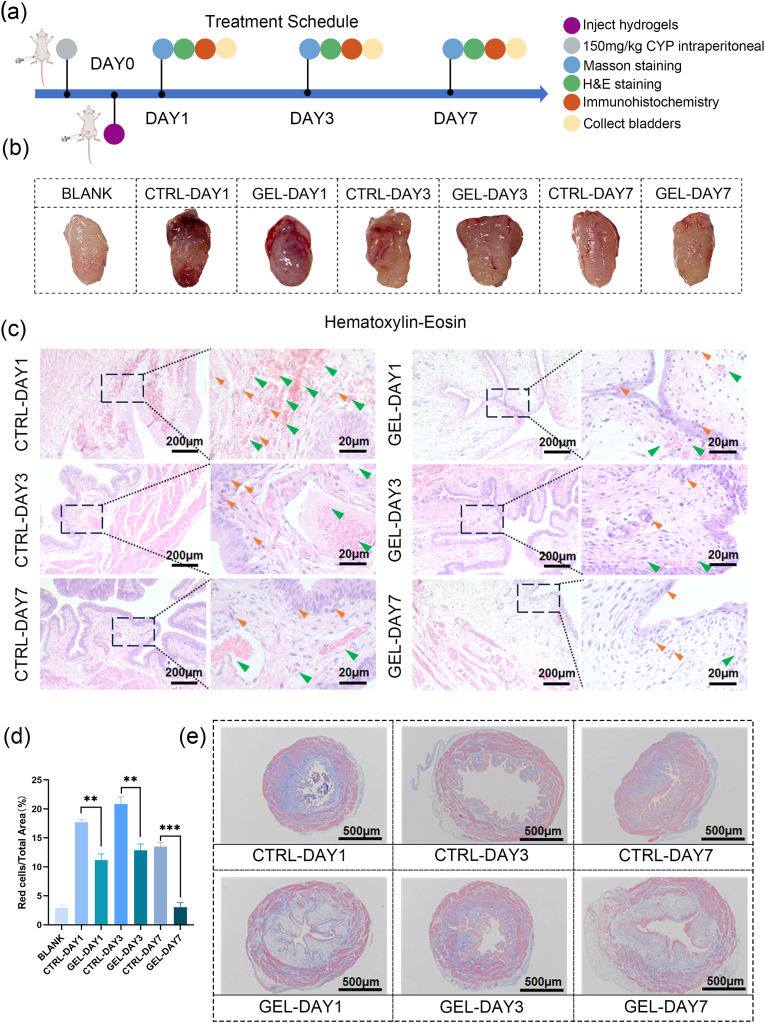
(a) Experimental schematic of CHMA/SFMA hydrogel adhesives assisted CHC bladder healing on SD rat models. (b) Photographs of CHC bladders with varied treatments on days 1, 3 and 7. BLANK: Blank; CTRL: Control; GEL: CHMA/SFMA hydrogel adhesives. (c) Representative H&E staining images of CHC bladders in CTRL and GEL groups on days 1, 3, and 7. Orange Arrows: inflammatory cell; Green Arrows: extravasation of erythrocytes. (d) Quantification of erythrocyte extravasation in H&E stained sections across different groups. **p < 0.01, ***p < 0.001. (e) Representative Masson Trichrome staining images of CHC bladders in CTRL and GEL groups on days 1, 3, and 7. (For interpretation of the references to color in this figure legend, the reader is referred to the Web version of this article.)

The CHMA/SFMA hydrogel adhesives exhibited convenient injectability, high wet-adhesion, robust cell-responsive growth, anti-inflammatory, and rapid hemostasis performances, making them highly suitable for CHC treatment. To investigate the potential beneficial effects of 2%CHMA/10%SFMA hydrogels in mitigating CYP-induced cystitis in vivo, we established a SD rat CHC model through intraperitoneal injection of 150 mg/kg CYP following the experimental plan (Fig. 6a). The 2%CHMA/10%SFMA hydrogels were injected into the bladder via a syringe, and the animal's position was flipped to uniformly distribute the hydrogel across over the bladder inner wall (Fig. S11, Supporting Information). Clinical manifestations were carefully observed, the rats were sacrificed on day 1, 3 and 7 with tissue of bladder collected. As shown in Fig. 6b, the bladders of CTRL group suffered severe damage on day 1, characterized by massive blood vessel rupture, erythrocyte extravasation, and ulceration. In contrast, the GEL group exhibited an obvious reduction in erythrocyte extravasation and bleeding.

More detailed bladder healing processes were investigated by histological analysis, including H&E staining and Masson Trichrome staining. As shown in Fig. 6c, on day 3, the CTRL group showed a high inflammation and erythrocyte extravasation, while the GEL group on day 3 exhibited milder bleeding and inflammation. By day 7, although both groups showed reduced bleeding and inflammatory cell infiltration, the GEL group displayed better recovery than the CTRL group. Additionally, the submucosal stromal layer was thicker in the GEL group than in the CTRL group on days 1, 3 and 7 (Fig. 6c and d, Fig. S12, Supporting Information). These results indicated that the hydrogel exerts a hemostatic and reparative effects on hemorrhagic cystitis. Masson Trichrome staining further revealed extensive collagen deposition in the GEL group (Fig. 6e, Fig. S13, Supporting Information). Furthermore, after 14 days of hydrogel treatment, the urine resumed a normal yellow color, and hematuria had completely disappeared, indicating successful relief from the symptoms of hemorrhagic cystitis (Fig. S14, Supporting Information).

**Fig. 7:**
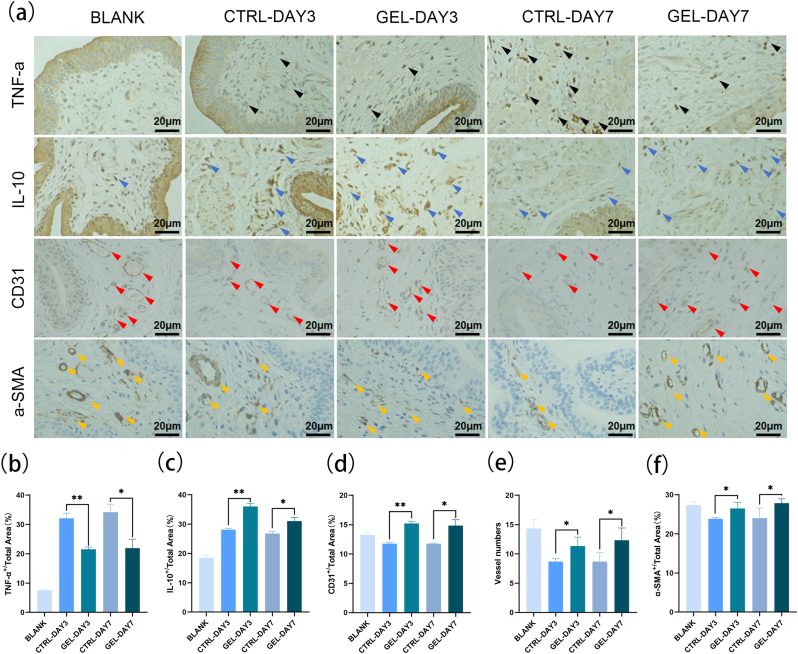
(a) Representative immunohistochemistry staining images of Blank and CHC bladders on days 3 and 7, including TNF-α, IL-10, CD 31, and α-SMA. (b–f) Percentage of positive TNF-α (b), IL-10 (c), CD 31 (d), vessel numbers (e), and α-SMA (f) within total bladder area. *p < 0.05, **p < 0.01.

Immunohistochemical analysis was further conducted to assess the healing process of CHC bladders. Inflammation response, angiogenesis, and smooth muscle regeneration are critical indicators of wound healing. Therefore, immunostainings for TNF-α, IL-10, CD31, and α-SMA were performed. As shown in Fig. 7a, the expression of TNF-α was significantly decreased in the GEL group compared with the CTRL group at the corresponding time points, while IL-10 expression increased in the GEL group than that in the CTRL group (Fig. 7b and c), indicating an inflammatory regulation effect of CHMA/SFMA hydrogel adhesives. Moreover, there was not only a significant difference in the expression level of CD31 but also a higher vessel number in the GEL group than in the CTRL group (Fig. 7a, d and 7e), indicating the promoting vascularization function of CHMA/SFMA hydrogel adhesives. Additionally, the α-SMA was more expressed in GEL group compared with the CTRL group (Fig. 7a and f), signifying the well-preserved structural integrity of muscle cells. These results demonstrated that CHMA/SFMA hydrogels promotes the tissue regeneration of bladders through regulating inflammatory effects and promoting angiogenesis.

## 4 Conclusion

In this study, we developed a novel injectable and biodegradable hydrogel adhesive by combining chitosan methylacryloyl (CHMA) with silk fibroin methylacryloyl (SFMA) to facilitate the healing of cyclophosphamide-induced hemorrhagic cystitis bladder injuries. Attributing to abundant non-covalent interactions like hydrogen bonding and electrostatic interactions, the optimized 2%CHMA/10%SFMA hydrogel precursors exhibited a pre-gel with shear-thinning and rapid self-healing, which allows for micro-injection during applications. Following a brief photo-polymerization within 10 s, the slightly crosslinked hydrogels demonstrated adequate mechanical strength and toughness suitable for bladder. They also exhibited and strong adhesion capabilities to bladder tissues (26.21 N/m), providing protection to the injured bladder from urine exposure. In simulated artificial urine, the slight crosslinked CHMA/SFMA hydrogel adhesives degraded within 16 days in-vitro and 14 days in-vivo. Leveraging the synergistic effect of CHMA and SFMA components, the hydrogel promoted the microaggregate growth of SV-HUC-1 in vitro and regulated macrophage polarization. The hydrogels also showed good hemocompatibility and rapid hemostasis performance, with a hemostatic time within 15 s and blood loss blow 135.4 mg. For potential CHC bladder treatment in SD rat models, the CHMA/SFMA hydrogel adhesives facilitated a programmed inflammation regulation, rapid angiogenesis, and myogenesis for CHC bladder healing. Overall, this innovative hydrogel adhesive holds great promise as a meaningful clinical alternative for CHC bladder repair.

## CRediT authorship contribution statement

**Jie Yao:** Conceptualization, Data curation, Formal analysis, Funding acquisition, Methodology, Validation, Visualization, Writing – original draft, Writing – review & editing. **Yaoqi Chen:** Data curation, Formal analysis, Investigation, Methodology. **Xiang Zhang:** Formal analysis, Investigation, Methodology. **Junfeng Chen:** Conceptualization, Funding acquisition, Validation. **Cheng Zhou:** Funding acquisition, Investigation, Validation. **Junhui Jiang:** Funding acquisition, Investigation, Project administration. **Hua Zhang:** Conceptualization, Data curation, Funding acquisition, Investigation, Methodology, Supervision, Writing – review & editing. **Kerong Wu:** Funding acquisition, Investigation, Project administration, Supervision, Writing – review & editing.

## Declaration of competing interest

The authors declare the following financial interests/personal relationships which may be considered as potential competing interests:Junhui Jiang reports financial support was provided by Ningbo Clinical Research Center for Urological Disease. Hua Zhang reports financial support was provided by Open Foundation of the 10.13039/501100011310State Key Laboratory of Fluid Power and Mechatronic Systems. Hua Zhang reports financial support was provided by Key Laboratory of Precision Medicine for Atherosclerotic Diseases of Zhejiang Province. Cheng Zhou reports financial support was provided by Zhejiang Natural Science Fund. Jie Yao reports financial support was provided by 10.13039/501100007928Ningbo Science and Technology Bureau. If there are other authors, they declare that they have no known competing financial interests or personal relationships that could have appeared to influence the work reported in this paper.

## Data Availability

No data was used for the research described in the article.
