# Supplementary material for: Slightly photo-crosslinked chitosan/silk fibroin hydrogel adhesives with hemostasis and anti-inflammation for pro-healing cyclophosphamide-induced hemorrhagic cystitis
Source: Mater Today Bio. 2024 Jan 5;25:100947. doi: 10.1016/j.mtbio.2024.100947 (PMC10826334; doi:10.1016/j.mtbio.2024.100947)
Supplement: Multimedia component 1 [file mmc1.docx]

**Supporting Information**

**Slightly Photo-crosslinked Chitosan/Silk fibroin Hydrogel Adhesives with Hemostasis and Anti-inflammation for Pro-healing Cyclophosphamide-induced Hemorrhagic Cystitis**

Jie Yao^a, d^, Yaoqi Chen^a^, Xiang Zhang^a^, Junfeng Chen^a^, Cheng Zhou^a^, Junhui Jiang^a^, Hua Zhang^b, c, d^*, Kerong Wu^a, d^*

1. Department of Urology, Translational Research Laboratory for Urology, Ningbo Clinical Research Center for Urological Disease, The First Affiliated Hospital of Ningbo University, Ningbo, Zhejiang 315010, China
2. State Key Laboratory of Fluid Power and Mechatronic Systems, Zhejiang University, Hangzhou, Zhejiang 310027, China
3. Research Institute of Smart Medicine and Biological Engineering, Ningbo University, Ningbo, Zhejiang 315211, China
4. Key Laboratory of Precision Medicine for Atherosclerotic Diseases of Zhejiang Province, The First Affiliated Hospital of Ningbo University, Ningbo, Zhejiang 315010, China

***Corresponding Authors**. fyywukerong@nbu.edu.cn; zhanghua@nbu.edu.cn.


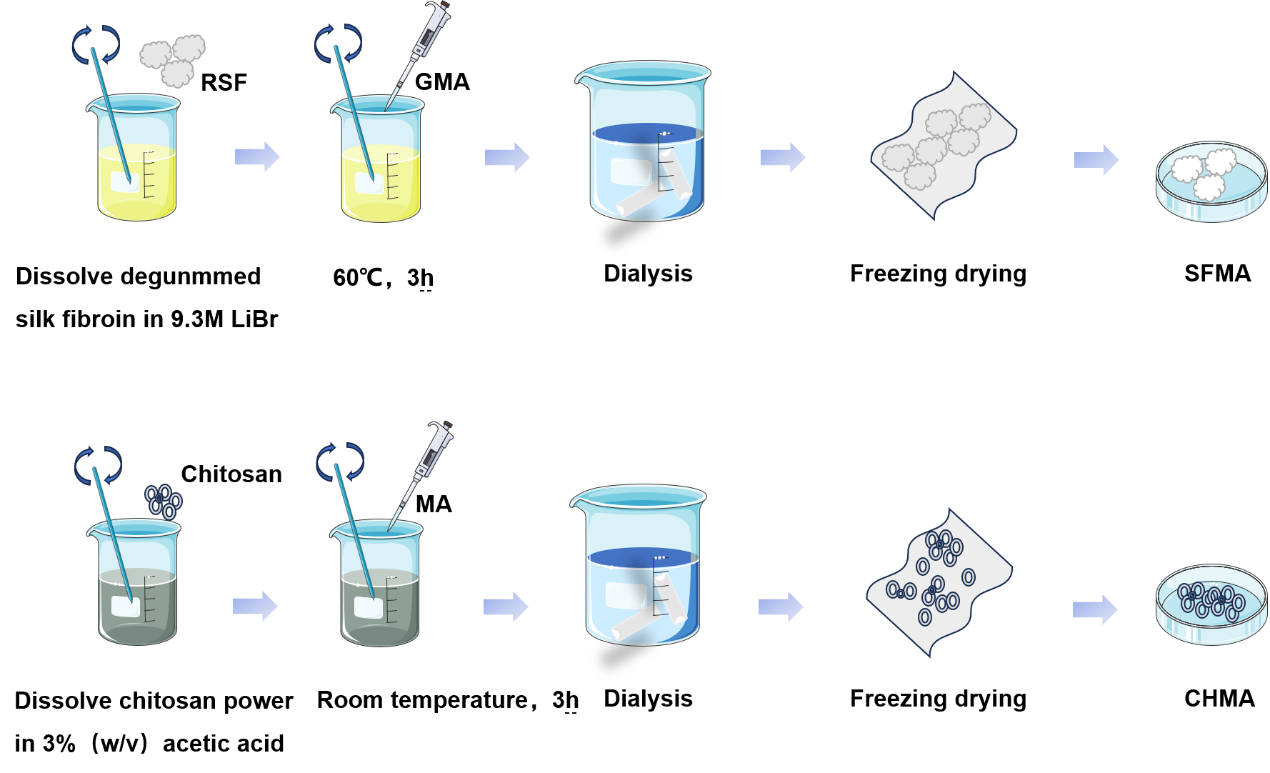


Fig. S1. Schematic of silk fibroin methylacryloyl (SFMA) and chitosan methylacryloyl (CHMA) synthesis.


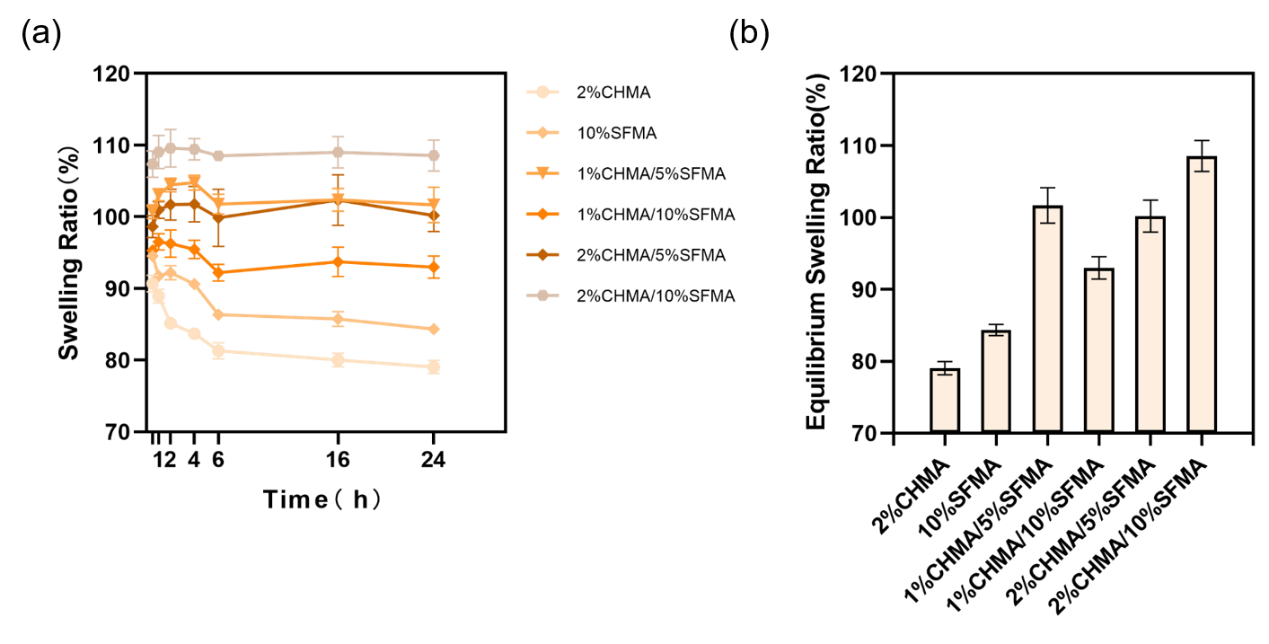


Fig. S2. (a) Swelling behavior and (b) equilibrium swelling ratio of CHMA/SFMA hydrogels with varying compositions in artificial urine.


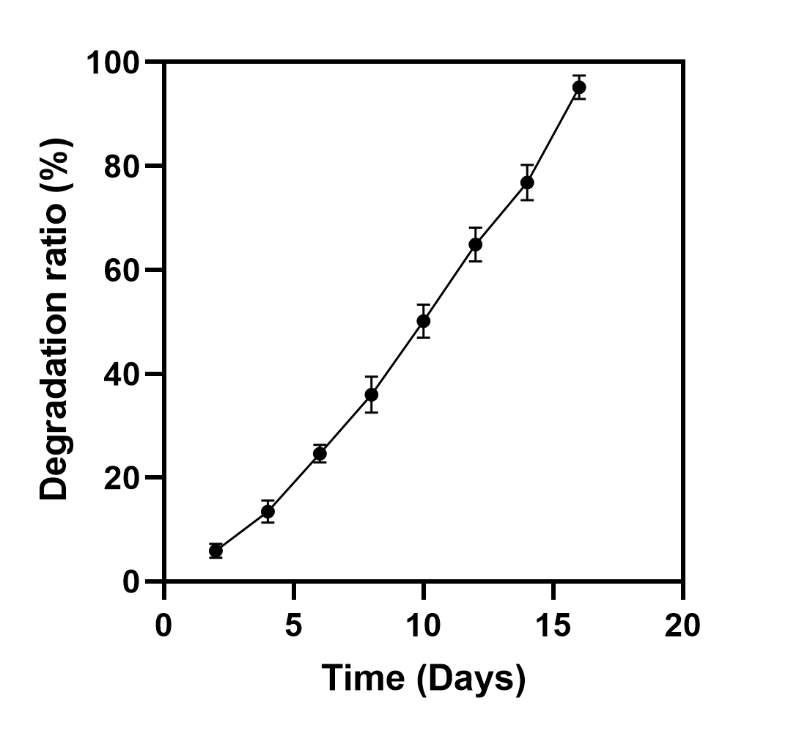


Fig. S3. Degradation ratio of 2%CHMA/10%SFMA hydrogels in artificial urine containing 0.02% sodium azide and 2 U/mL collagenase type I.


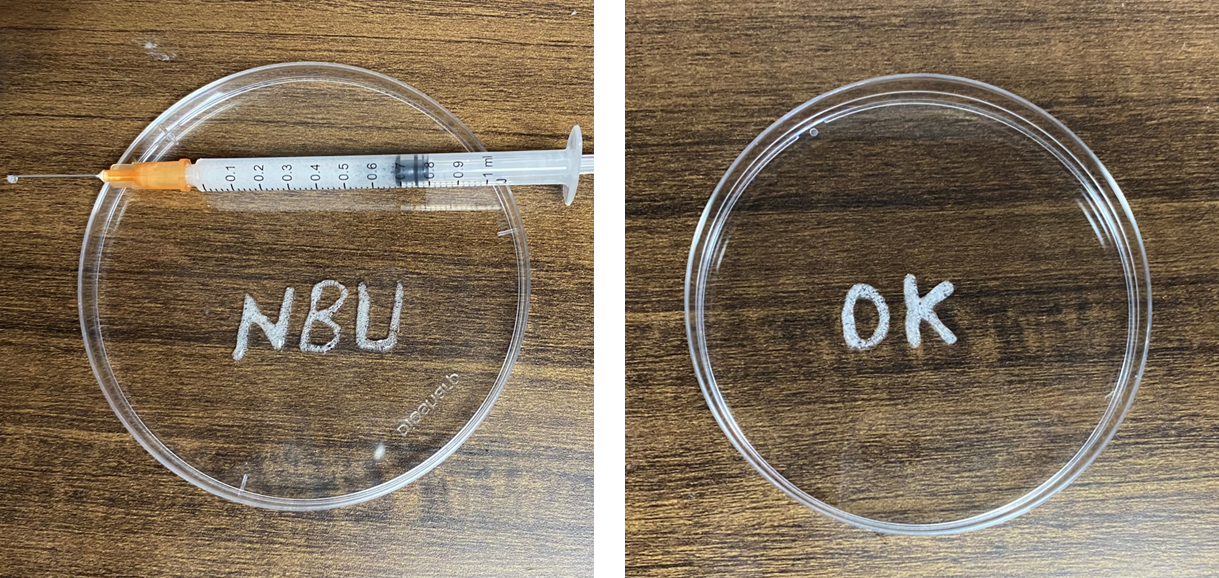


Fig. S4. The injectability of 1%CHMA/10%SFMA pregels.


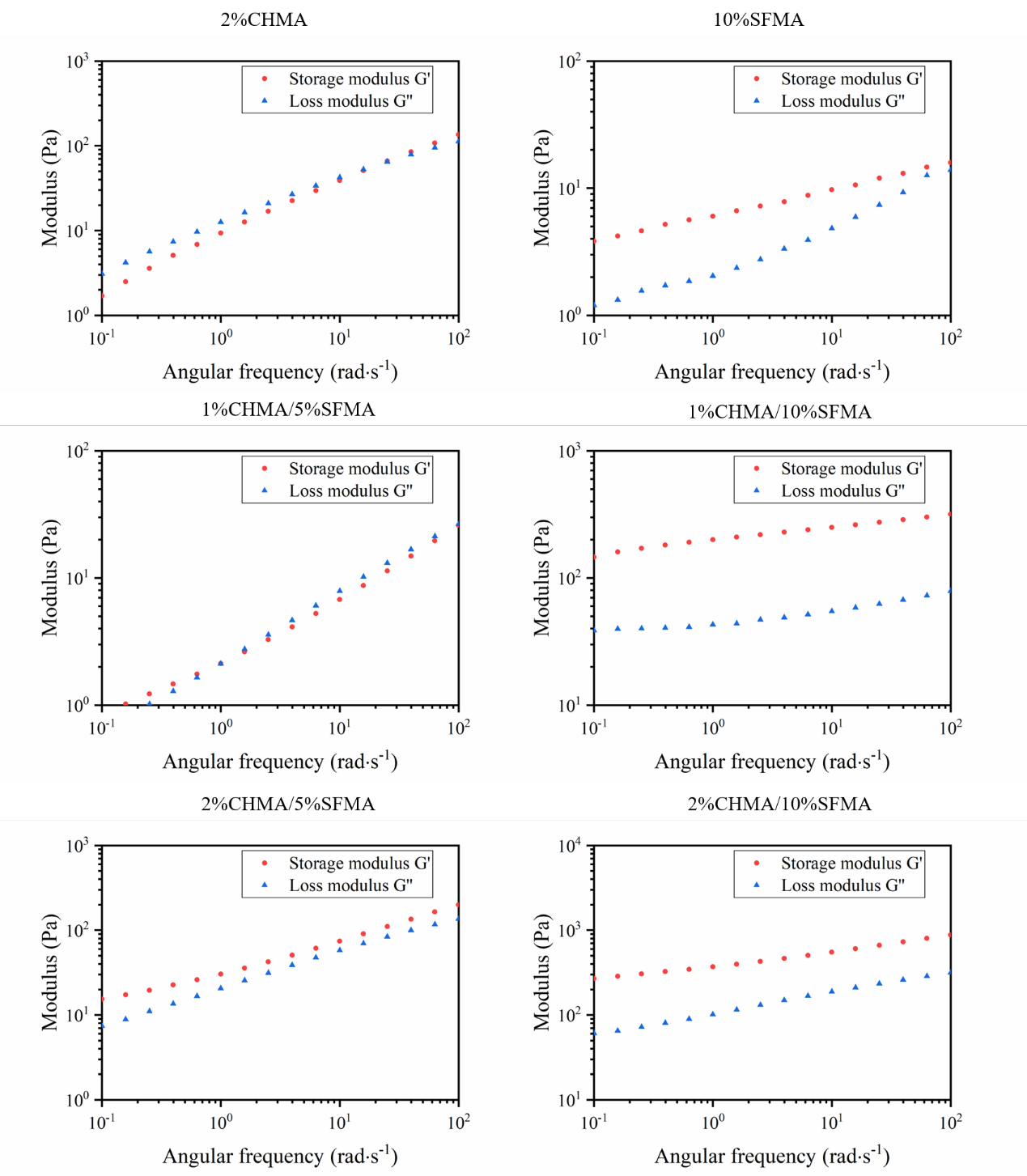


Fig. S5. Frequency sweeps of CHMA/SFMA hydrogel precursors with varying compositions, illustrating a gel-like behavior, except for the pure CHMA and SFMA solutions.


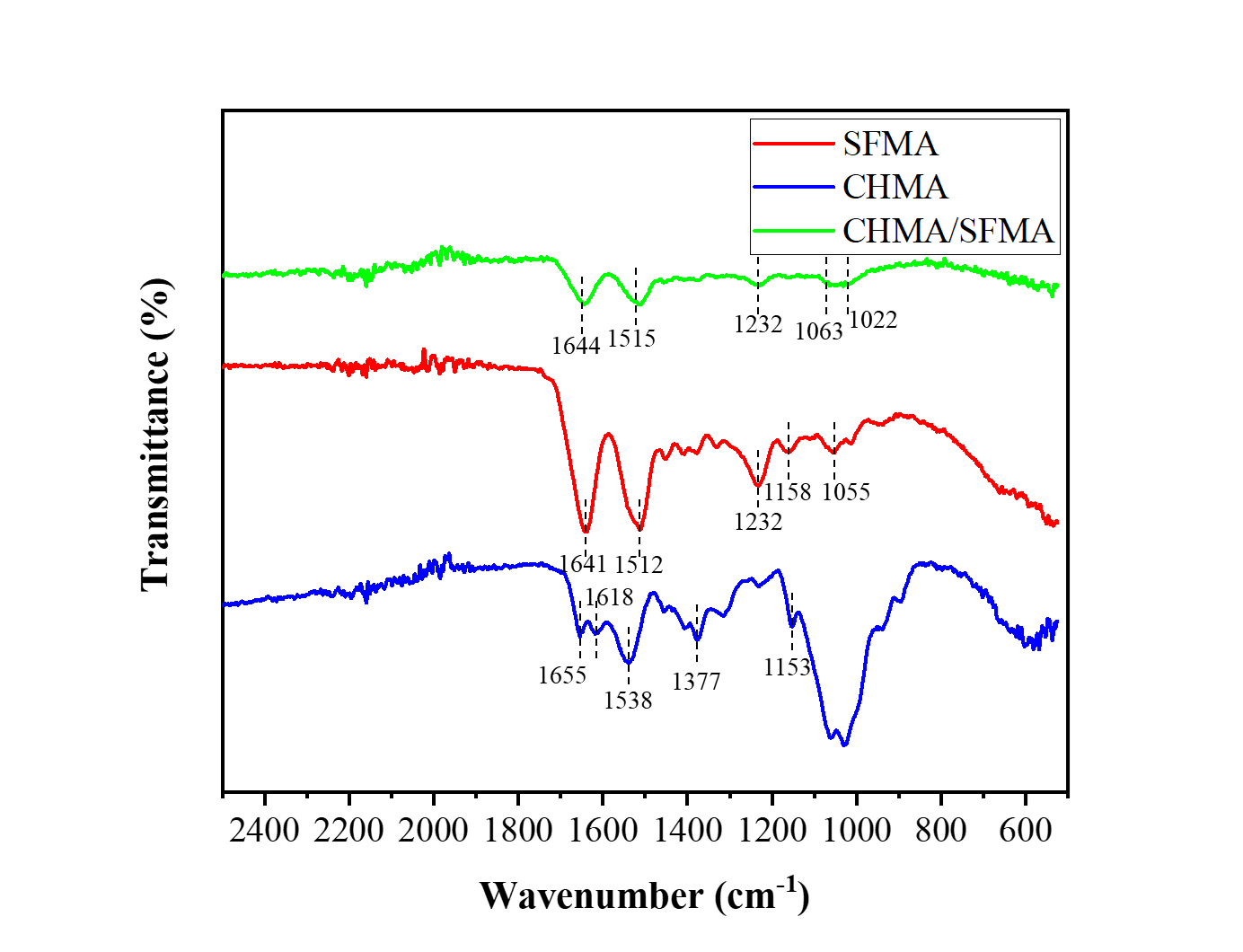


Fig. S6. FT-IR spectra of SFMA, CHMA, and photo-crosslinked CHMA/SFMA hydrogel.

Table S1. Polymer network pore sizes of hydrogels analyzed using rubber elastic theory.

| Group | G (Pa) | V (nm^3^) |
| --- | --- | --- |
| 2%CHMA | 6.75 | 6.09×10^5^ |
| 10%SFMA | 9.71 | 4.24×10^5^ |
| 1%CHMA/5%SFMA | 6.75 | 6.09×10^5^ |
| 1%CHMA/10%SFMA | 250.07 | 1.64×10^4^ |
| 2%CHMA/5%SFMA | 74.38 | 5.53×10^4^ |
| 2%CHMA/10%SFMA | 551.24 | 7.46×10^3^ |


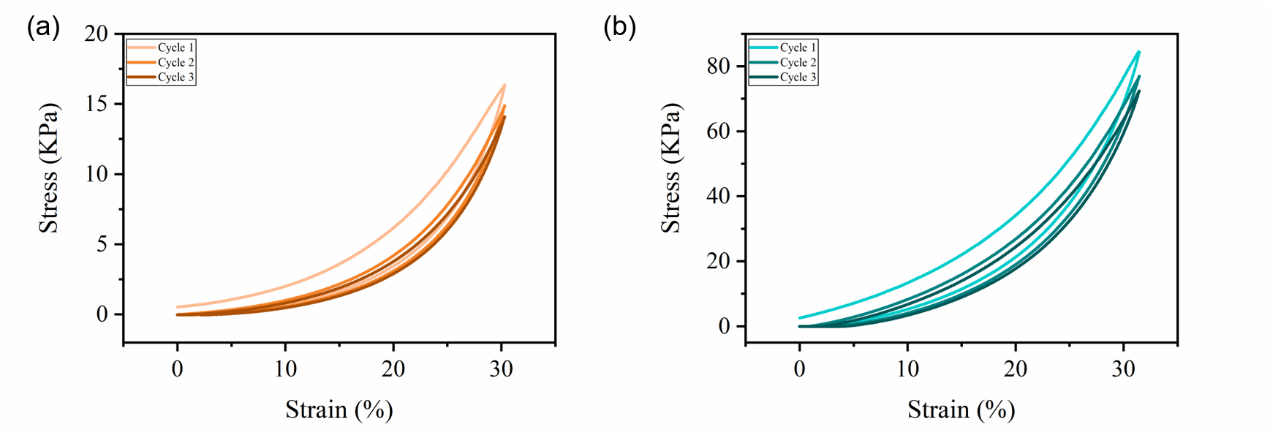


Fig. S7. Cyclic compression test of the (a) 1%CHMA/10%SFMA and (b) 2%CHMA/10%SFMA hydrogels under three repeated cycles of up to 30% strain.


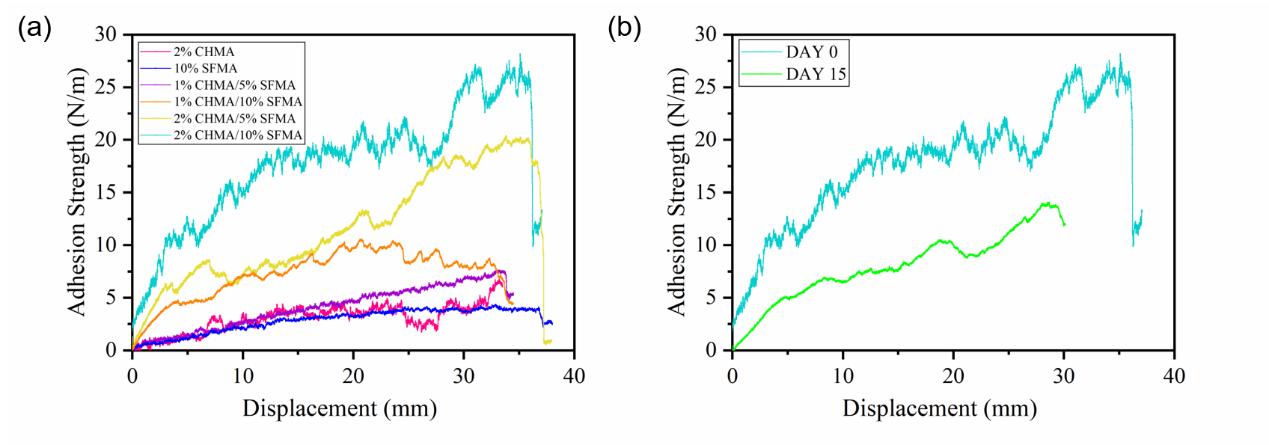


Fig. S8. (a) Representative curves of the 90-degree peel test between porcine bladder and hydrogels. (b) 2%CHMA/10%SFMA hydrogels measured directly on day 0 and after 15 days of immersion.


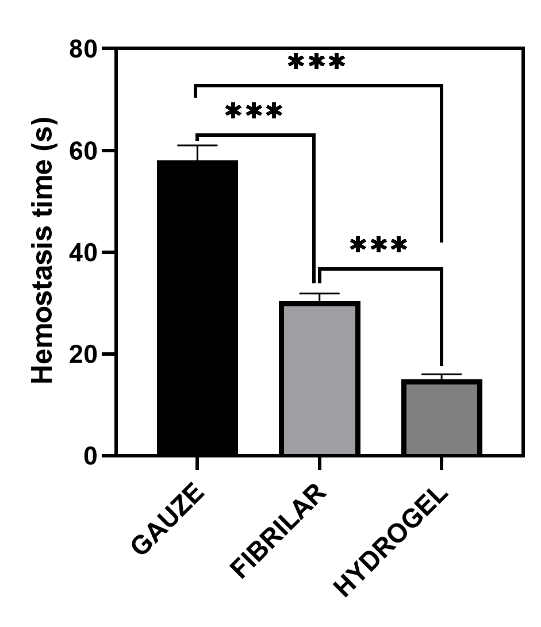


Fig. S9. The hemostasis time of Gauze, surgicel® fibrillar™ and 2%CHMA/10%SFMA hydrogels. ****p* < 0.001.


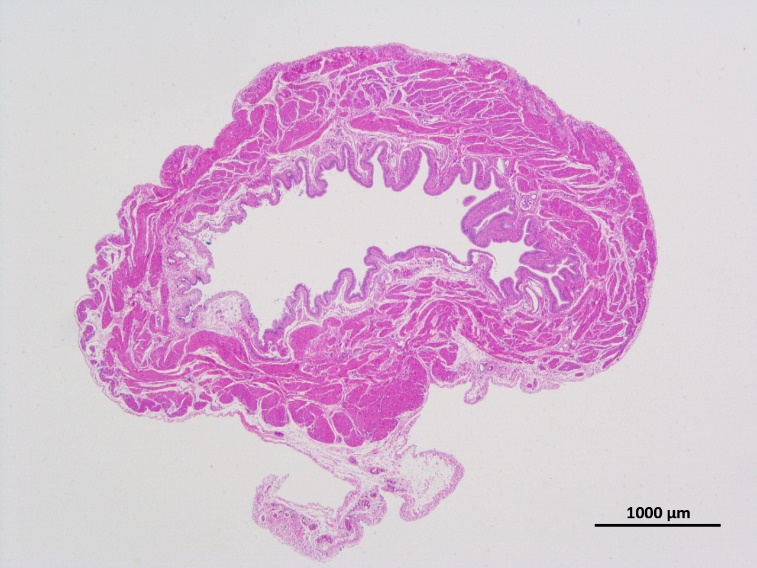


Fig. S10. Representative H&E staining images of the bladder on day 14 after treatment with 2%CHMA/10%SFMA hydrogel.


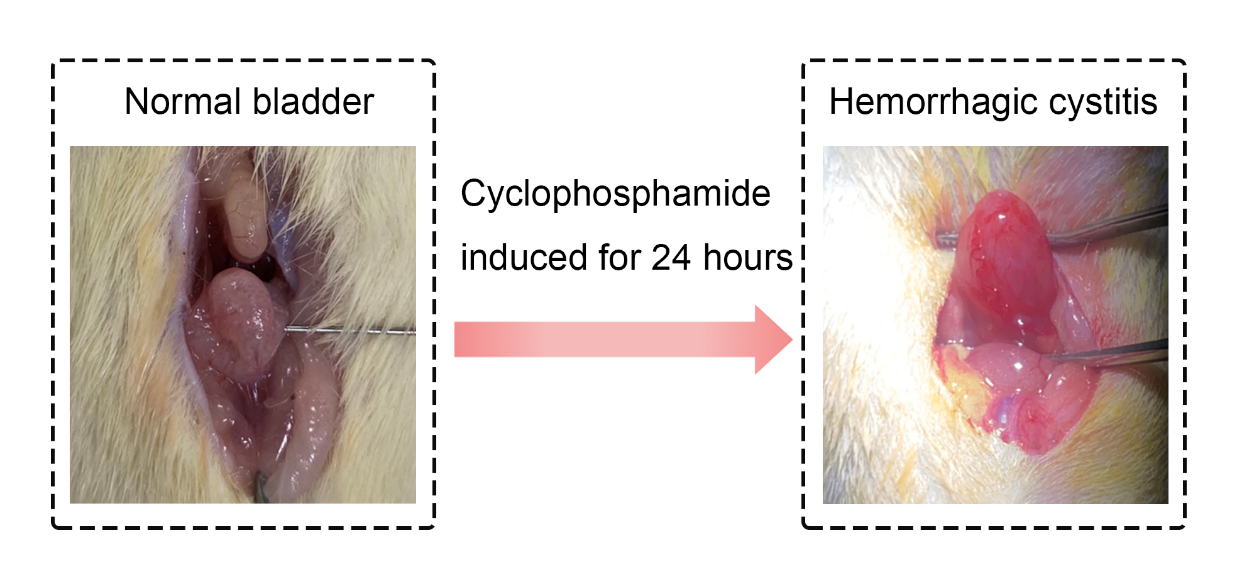


Fig. S11. An animal model of hemorrhagic cystitis bladder was established. A comparison photograph of normal and hemorrhagic cystitis bladders.


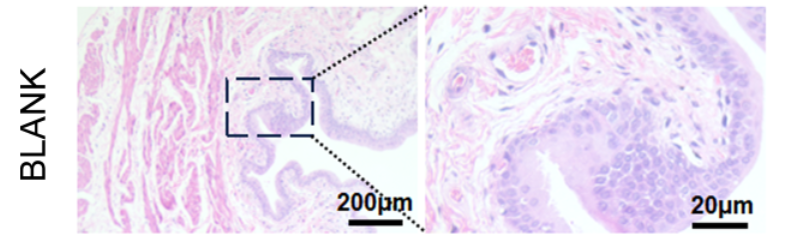


Fig. S12. Representative H&E staining images of blank bladders.


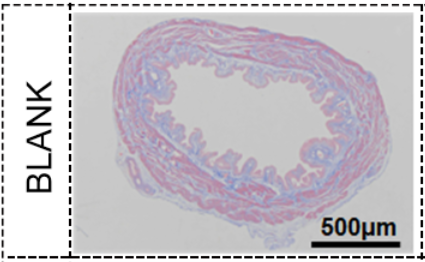


Fig. S13. Representative Masson Trichrome staining images of blank bladders.


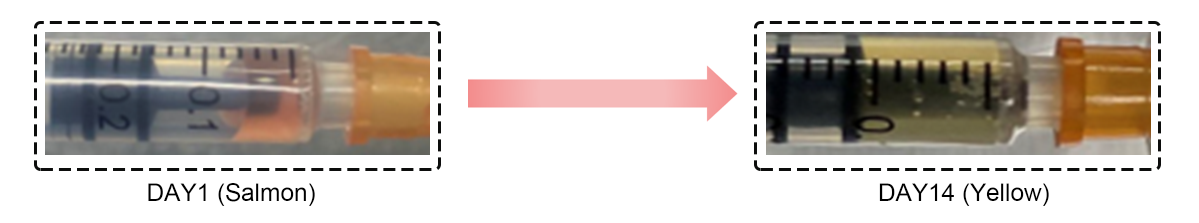


Fig. S14. Hematuria condition of DAY1 (Color. Salmon) and DAY14 (Color. Yellow).
